# Resolving deconvolution ambiguity in gene alternative splicing

**DOI:** 10.1186/1471-2105-10-237

**Published:** 2009-08-04

**Authors:** Yiyuan She, Earl Hubbell, Hui Wang

**Affiliations:** 1 Department of Statistics, Florida State University, Tallahassee, FL 32306-4330, USA; 2 Affymetrix Inc, Santa Clara, CA 95051, USA; 3 Veracyte Inc, South San Francisco, CA 94080, USA

## Abstract

**Background:**

For many gene structures it is impossible to resolve intensity data uniquely to establish abundances of splice variants. This was empirically noted by Wang *et al*. in which it was called a "degeneracy problem". The ambiguity results from an ill-posed problem where additional information is needed in order to obtain an unique answer in splice variant deconvolution.

**Results:**

In this paper, we analyze the situations under which the problem occurs and perform a rigorous mathematical study which gives necessary and sufficient conditions on how many and what type of constraints are needed to resolve all ambiguity. This analysis is generally applicable to matrix models of splice variants. We explore the proposal that probe sequence information may provide sufficient additional constraints to resolve real-world instances. However, probe behavior cannot be predicted with sufficient accuracy by any existing probe sequence model, and so we present a Bayesian framework for estimating variant abundances by incorporating the prediction uncertainty from the micro-model of probe responsiveness into the macro-model of probe intensities.

**Conclusion:**

The matrix analysis of constraints provides a tool for detecting real-world instances in which additional constraints may be necessary to resolve splice variants. While purely mathematical constraints can be stated without error, real-world constraints may themselves be poorly resolved. Our Bayesian framework provides a generic solution to the problem of uniquely estimating transcript abundances given additional constraints that themselves may be uncertain, such as regression fit to probe sequence models. We demonstrate the efficacy of it by extensive simulations as well as various biological data.

## Background

Alternative splicing is a ubiquitous phenomenon among eukaryotic organisms. Based on the most recent studies, more than 74% of the human genes with multiple exons undergo this process [1]. Alternative splicing plays an important role in the function of eukaryote organisms. It is a versatile process that can be integrated with other regulatory mechanisms to generate protein diversity, to modulate cellular responses, and to regulate biochemical pathways [2-15]. Changes in splicing are often associated with genetic disease and cancer [16-25]. Thus being able to measure and compare transcript variant abundance is critical for understanding the function of a gene.

Many studies have been taken to perform genome-wide analysis of alternative splicing using microarrays. Most current approaches, however, focus mainly on qualitatively detecting splicing events [1,26-33] by examining the presence or absence of known variant-specific exons or junctions. Presence or absence of a variant is then inferred based on the transcript annotation.

On the other hand, quantitatively measuring gene-level expression has been a key task in microarray analysis (see, for example [34,35]). Most current methods, however, impose a simple identifiability constraint on *probe responsiveness *(often confused with chemical affinity, despite depending on multiple factors including hybridization affinity, which may play a part in the poor prediction accuracy of pure sequence models) for each probe set (such as individual exons). Accordingly, none of these algorithms can reconstruct all variant abundances (across exons, or between gene families) on the same scale; though perhaps not a problem when comparing the estimates across experiments, it causes insurmountable difficulty when attempting to compare among different variants. Further, even concentrating on a single family of variants, the mathematical linkage between intensity and the multiplicative model allows for ambiguous reconstruction. The aim of this paper is to find a generic method to estimate variant abundances consistently for all variant structures.

We first review the mathematical model and prove necessary and sufficient conditions to detect and resolve ill-posed instances of the model.

In a previous work, Wang *et al*. [36] have implemented a model-based method to estimate the relative concentrations of known splice variants by incorporating gene structure into a probe intensity model [34], where the probe intensity is modeled by probe responsiveness and probe abundance, plus an error term. The gene structure specifies the relationship between known transcript variants, genomic features and probes, where genomic features can be either exons or junctions. This linkage enables them to use a probe intensity model to estimate the relative abundance of variants. Maximum likelihood estimation (MLE) is used to obtain the estimates for variant concentrations and probe affnities.

**Figure 1:**
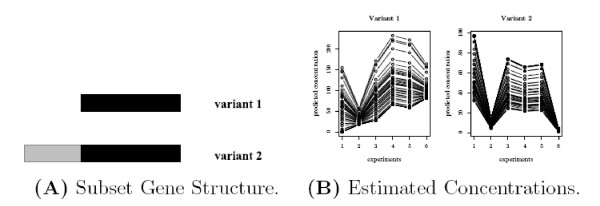
**Subset gene structure and the corresponding estimated concentrations**. (A) shows a 2-variant "subset" gene structure, where the genomic composition of variant 1 is a subset of variant 2. (B) shows the estimated concentrations of the two variants for 20 different initial values using Wang *et al*.'s method [36]. Each line indicates estimated concentrations with one set of initial value; all are globally optimal solutions that give the same RSS. See [36] for details.

**Figure 2:**
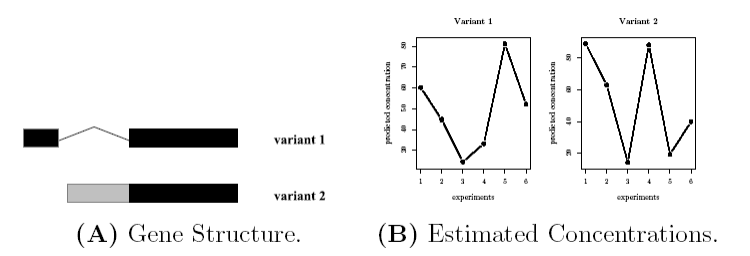
**A simple two-variant gene structure and its estimated concentrations using Wang et al.'s method **[36]. (A) shows a simple two-variant gene structure with three exons. The two variants have one common exon and each variant contains one unique exon. (B) shows the estimated concentrations with 20 different initial values. The solution is unique for the two variants.

This method often leads to unresolved ambiguities in reconstruction. We have observed the algorithm can not produce a unique solution for many gene structures [36]. The MLE gives multiple solutions, each one attaining the (globally) optimal function value. A simple example is the so-called "two-variant subset" gene structure, where the feature composition of one variant is a subset of the other one. Biologically, if the two variants of a gene use different promoters or 3' UTRs, the gene structure will display the "subset" property. Figure 1B shows the simulation output for such a gene structure given in Figure 1A. The algorithm generates different estimates for probe responsiveness and variant concentration in different runs even though the residual-sum-of-squares (RSS) are the same. (For comparison, a simple gene structure that doesn't have this problem is shown in Figure 2. The algorithm always converges to a unique solution.) We have also observed that the same issue widely exists in more complex gene structures.

This is an example of an "ill-posed" problem, where additional constraints must be added to overcome the singularity, otherwise the ambiguity will seriously limit the usage of the above method. More recently, Shai *et al*. [37] developed a probability model using unsupervised Bayesian learning to estimate variant concentrations. Some constraints are obtained in a "semi-supervised" fashion by minimizing the root mean squared error between the final predictions and the RT-PCR results. Unfortunately, their model will encounter the same nonuniqueness problem in general. However, as a special case, for their cassette exon example with only two variants and the correspondingly simple gene structure (shown in Figure 2A), there is no ill-posed issue and the solution is unique.

The aim of this paper is to find a generic method to estimate variant abundances consistently for all variant structures. We first review the mathematical model and prove necessary and sufficient conditions to detect and resolve ill-posed instances of the model. The natural source of information contained in probe sequences is used to construct additional constraints in the deconvolution. Due to the great difficulty of predicting probe effects via sequence composition, this probe sequence based model does not produce mathematically perfect constraints. We therefore incorporate a probe responsiveness model into a supervised Bayesian framework, which can accommodate imperfect or imprecise constraints. A useful side effect is that this model allows for reconstructing all variant abundances on the same scale between exons, and further, if the probe affinity model can be improved to produce sufficiently accurate predictions, reconstruct abundances on the same scale between genes. We demonstrate the potential efficacy of this method through simulation as well as experimental data. Although the overall success in recovering comparable signal values across genes is not yet great, our model succeeds in uniquely resolving alternative splicing data even given the loose constraints from an imprecise model.

## Results

We first demonstrate the effectiveness of our Dulmage-Mendelsohn decomposition based grouping procedure on some simulation data. Then the performance of our Bayesian method is shown on two real biological datasets. As we shall see, although the overall fitness on ***T ***is not yet good enough, we do succeed in recovering important quantities none of the other methods can. R and Matlab [38] were used.

### Simulation Data

In this subsection, we use simulation data with no sequence information to demonstrate the effectiveness of the grouping procedure in removing the non-uniqueness during transcript deconvolution. Deriving the grouping procedure and building probe responsiveness models, though related to each other in the framework, are two independent tasks. By using simulation data, we can skip the errors in a probe responsiveness model built on probe sequences. This follows the case in which we have perfect knowledge of the response of each probe (the limiting case of a sequence model). We have tried large amount of data with varying sizes and different gene structures. One such example is presented as follows.

In this simulation dataset, there are 5 probesets, each one containing 2 probes, 4 variants, and 6 experiments. So the probe intensities ***Y ***is a 10-by-6 matrix. For space reasons, we only list the gene structure matrix ***G ***here:



In addition to the Gaussian noise ~ *N*(0,100) added to the true signal (***AFGT***), we appended a significant background signal uniformly drawn from [1, 200] to increase the deconvolution difficulty. This background signal is completely unknown in the estimation. Our task is to reconstruct both ***T ***and ***A ***from ***Y ***with ***F ***and ***G ***known.

**Figure 3:**
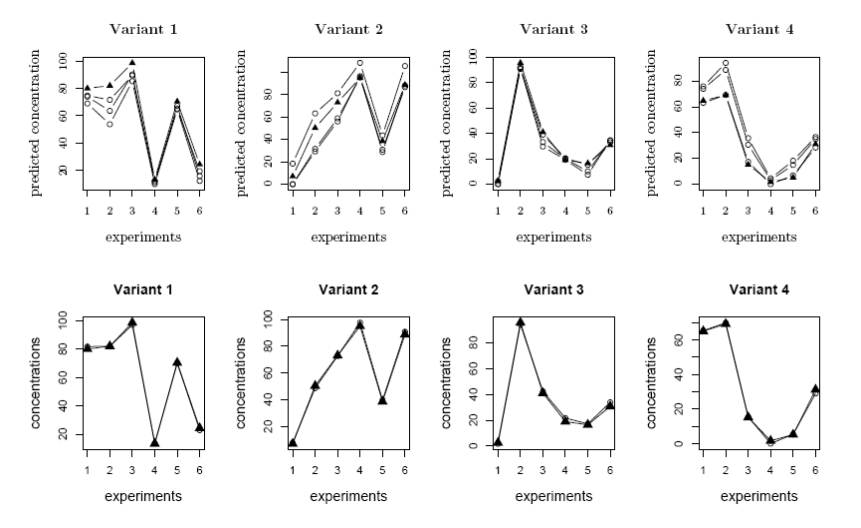
**Predicted ***T ***on simulation data without and with group constraints**. In the upper panel, we plotted the estimated concentrations of the three transcripts generated by Wang *et al*.'s deconvolution procedure without group constraints [36] (denoted by the circles). Note that every solution achieves a global minimum of the log-likelihood function, but they are quite different and none of them approximates the true ***T ***(denoted by the triangles). The lower panel shows the *unique *estimate using (four) group constraints; it is very close to the true ***T***, even though the noise is large and there is some unknown background signal.

With no grouping and no additional constraints, the solutions are not unique (infinitely many, as a matter of fact) and each one is a global minimum of the log-likelihood but not the truth. See the upper panel of Figure 3 for an illustration. Although it is relatively easy to get a globally optimal solution, there is no appropriate criterion to tell which estimate is correct or gives the best approximation.

Applying the grouping procedure described in the **Methods **Section below to this data, we obtain four probe groups, which suggests a high deconvolution singularity. Group constraints are then constructed from the true ***A ***or ***T ***to help fit the model. We use alternative optimization with group-rescaling to solve the model. The lower panel of Figure 3 shows that after adding the group constraints in the fitting procedure, the prediction matches perfectly with the true answer, even with some substantial unknown background signal.

### Biological Data

In this subsection, we demonstrate the power of our general framework with two biological datasets – HG-SV and HG-LS.

#### Data description

Two data sets are used in this paper to train the sequence model and estimate the concentrations.

1. Human gene Latin-square (HG-LS) data. The data can be found at affymetrix's web site at . This Latin Square design consists of 14 spiked-in gene groups in 9 experimental groups. The concentrations of the 7 gene groups in the first experiment are 0, 0.25, 0.75,1.5,3,12,24,192 and 386 pM. Each subsequent experiment rotates the spike-in concentrations by one group, i.e., experiment 2 begins with 0.25 pM and ends at 0 pM, on up to experiment 7, which begins with 386 pM and ends with 192 pM. Each experiment contains 3 replicates. There are 26 probe sets and 286 PM/MM probe pairs in total.

2. Human gene splice variants (HG-SV) data. The data was generated using a custom designed array. Please refer to [36] for details of array design and experimental design. Four genes (MYLK, TPM2, CD44, MAPT) with two clone variants were spiked in with varying concentrations. We first mixed targets derived from two variants (clone 1 and clone 2) with differing concentrations: the first variant ranged from 0 to 64 pM and the second variant ranged from 64 pM to 0 pM with the total concentration held constant at 64 pM. By diluting the whole set 4 and 16 times, we obtained further results for titration sets with total concentrations of 16 pM and 4 pM respectively. There are 42 exon and exon/exon junction probesets and 330 PM/MM probe pairs in total. The sequences of these 8 variant clones are all known.

#### HG-SV data

**Figure 4:**
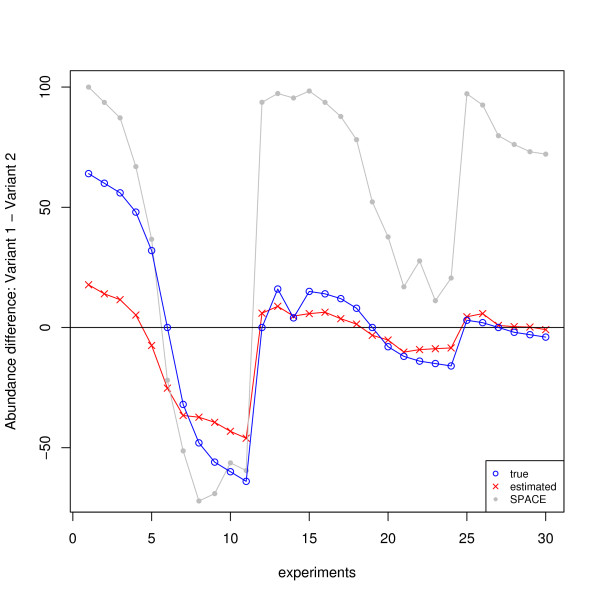
**The concentration differences between variant 1 and variant 2 for MAPT (HG-SV data): predicted vs. true**. The predicted concentration differences and the true differences are labeled by red crosses and green circles respectively. For comparison purpose, the estimates using the SPACE algorithm [39] are also plotted, represented by gray dots.

The HG-SV data described previously have two known transcript variants in their gene structure. In this experiment, we took genes MYLK and TPM2 as training data (125 probes in total), CD44 as validation data (128 probes), and MAPT (77 probes) as test data. The unknown ***T ***is of size 2-by-30, with 2 variants and 30 experiments. On this relatively small data set, the estimate is, inevitably, not perfect, with the median absolute error (AE) given by 4.6, and the mean AE 8.8. However, all the ***T***-entries are estimated on the same scale and we do observe that our reconstruction is good in comparing the concentrations between the two variants. As demonstrated in Figure 4, our estimated concentration differences roughly follow the right trend for the 30 experiments. In particular, the obtained zero-crossings are approximately correct. For comparison purpose, we ran the SPACE algorithm and got much larger errors; the median and mean AE are 22.4 and 34.9 respectively. The SPACE estimates are much worse especially for the last 19 experiments, as clearly shown in Figure 4.

#### HG-LS data

Genes in HG-LS data set only have one transcript. Our framework is general enough to be applied to such data as well, corresponding to a special case where ***G ***= ***I***. Our goal here is to compare transcript abundance level between genes. Since the ***G ***matrix is an identity matrix, the number of additional constraints required for estimating all transcript concentrations on the original scale is equal to the number of transcripts. They can be constructed via probe responsiveness predicted by the sequence model (10). To evaluate the performance of the algorithm on this dataset, we used 12 transcripts for training, 7 for validation, and the remaining 7 for testing purposes.

Our method allows us (for the first time) to estimate the whole concentration matrix ***T ***with no ambiguity. The Bayesian method including the standard error information of the predicted responsiveness can handle a poor probe sequence model and is efficient. It is not yet satisfactory enough to recover every concentration precisely, with the mean AE given by 34.6 and the median 10.2. This is not so surprising considering the poor fit of current probe sequence-based models and the small sample size (286 probes in total) of the data. We also ran the SAPCE algorithm [39] which yields larger errors; the mean and median AE are 73.1 and 12.0 respectively.

On the other hand, it is interesting and encouraging to note that the between-experiment and between-variant information has been restored to a large extent using our method. To clearly show this, we compare the following three types of ratios, where ***T***(*i*,:) and ***T***(:, *j*) are used to denote the *i*-th row (or variant) and the *j*-th column (or experiment) of ***T***, respectively, and diag(***T***), adiag(***T***) to denote the diagonal and anti-diagonal of ***T***, and 'med' represents the median operator.

a. Across-experiment accuracy.

For each variant i, compare (*i*,:)/med((*i*,:)) to ***T***(*i*,:)/med(***T***(*i*,:)).

b. Across-variant accuracy.

For each experiment *j*, compare (:, *j*)/med((:, *j*)) to ***T***(:, *j*)/med(***T***(:, *j*)).

c. Across-variant-and-experiment accuracy.

Compare diag()/med(diag()) to diag(***T***)/med(diag(***T***)), and compare adiag()/med(adiag()) to adiag(***T***)/med(adiag(***T***)).

**Figure 5:**
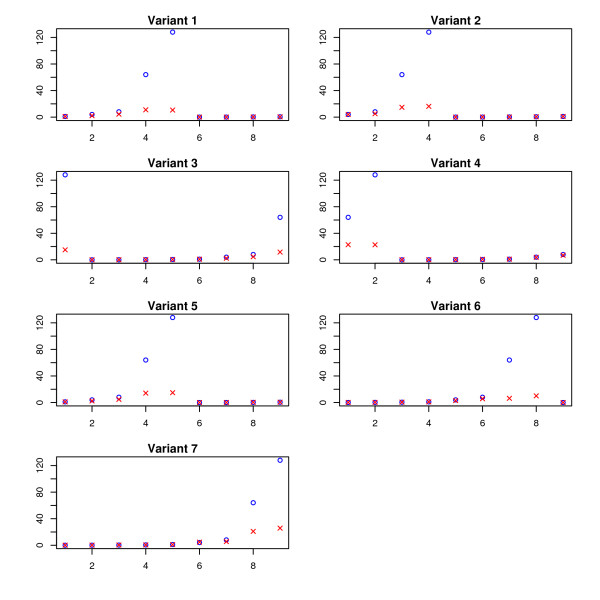
**Across-experiment ratios (on HG-LS data)**. The estimated values are denoted by crosses, while the true concentration ratios are denoted by circles. For each of the seven variants, we compare the across-experiment quantities (defined in (a) in the subsection of *HG-LS Data*) between the estimated  and the true ***T***.

**Figure 6:**
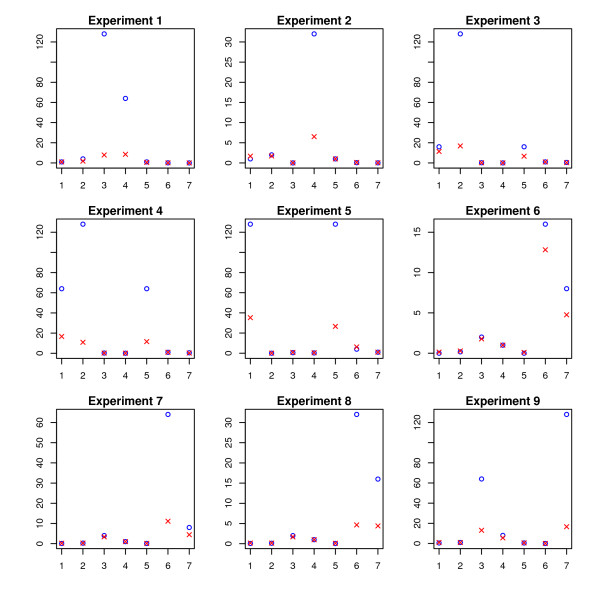
**Across-variant ratios (on HG-LS data)**. The estimated values are denoted by crosses, while the true concentration ratios are denoted by circles. For each of the nine experiments we compare the across-variant quantities (defined in (b) in the subsection of *HG-LS Data*) between the estimated  and the true ***T***.

**Figure 7:**
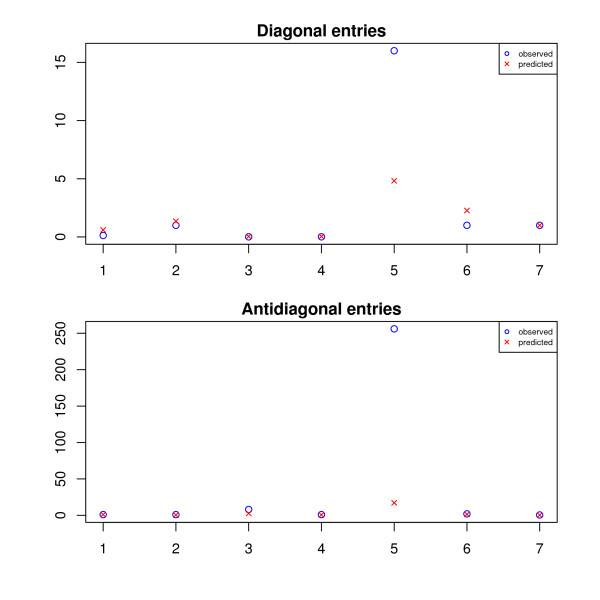
**Across-variant-and-experiment ratios (on HG-LS data)**. The estimated values are denoted by crosses, while the true values are denoted by circles. We compare the diagonal and antidiagonal (defined in (c) in the subsection of *HG-LS Data*) between the estimated  and the true ***T***.

**Figure 8:**
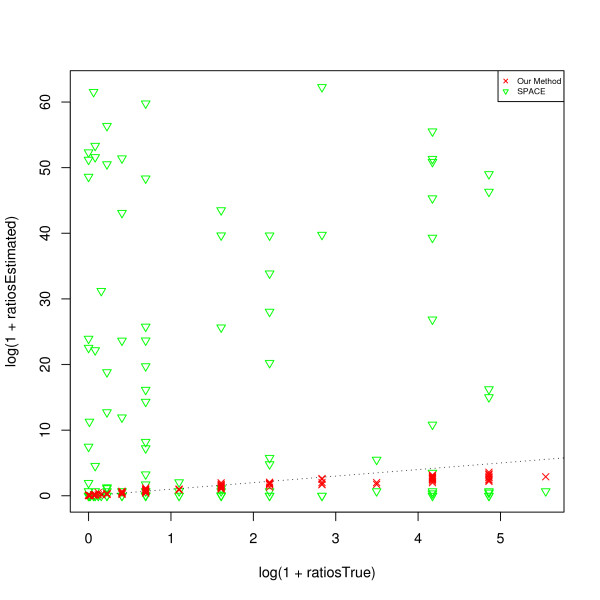
**A summary of all ratio comparisons (on HG-LS data)**. The *x *axis represents the true ratios of all three types (defined in the subsection of *HG-LS Data*); the *y *axis represents the results from our method denoted by red crosses, and from the SPACE algorithm by Anton *et al*. denoted by green triangles. The black dotted line is the identity line.

The results are plotted in Figure 5, Figure 6, and Figure 7. The SPACE estimates are poor and not plotted in these figures. To get an overall intuition, see Figure 8 for a summarized ratio-comparison of our method and the SPACE algorithm. One may find that our results make much more sense and are much closer to the truth especially for moderate ratios. By contrast, SPACE is not able to resolve all deconvolution ambiguities; its ***T***-estimate gives very misleading between-entry ratios. In all of the figures, we clearly see that our method is capable of restoring the ratios between the entries in ***T ***to some extent (either column-wise, row-wise, or diagonal-wise) except for very large ones. The lower predicted concentrations are likely due to experimental error where the variants were spiked in a lower concentration than specified (personal communication), as was noted in Wang *et al*. [36]. Our complete recovery of ***T ***is superior to single row recovery as most other methods are trying to solve. In particular, the across-row information can be very useful for comparing between different transcripts.

## Discussion

The singularity problem arising in deconvolution is common, both at the variant level and at the transcript level, if we want to recover from probe intensities all concentrations on the same scale. Our mathematical analysis and the grouping procedure in this paper apply to any complex gene structures in both situations. Furthermore, we built a Bayesian framework that adapts to any probe responsiveness model and probe intensity model (including the multiplicative-error models). It reduces the risk of using poorly predicted probe responsiveness based on current probe sequence models. Experimental results are positive and encouraging: our approach is able to reconstruct all variant abundances on the same scale, and thus for the first time allows for quantitatively comparing the estimated abundances between different transcripts. This is an advantage over most other methods which explicitly or implicitly impose an identifiability constraint on probe affinities for each probe set and are thus only meaningful for comparing the abundances across experiments.

In the experiments, our model cannot yet provide an adequate fit for the microarray data to reconstruct all concentrations precisely. A careful study shows that this is largely due to the poor fitness of existing probe sequence models (to-date) and the small number of probes of these datasets. We expect to develop a more accurate biophysics model to characterize the probe responsiveness and try our method on some larger microarrays as well. Moreover, since it is usually true that many probes might behave badly, further study is needed to investigate multiplicative probe intensity models and to design an appropriate robust fitting criterion in our Bayesian deconvolution framework.

## Conclusion

The matrix analysis of constraints provides a tool for detecting real-world instances in which additional constraints may be necessary to resolve splice variants. While purely mathematical constraints can be stated without error, real-world constraints may themselves be poorly resolved. Our Bayesian framework provides a generic solution to the problem of uniquely estimating transcript abundances given additional constraints that themselves may be uncertain, such as regression fit to probe sequence models. We demonstrate the efficacy of it by extensive simulations as well as various biological data.

## Methods

We will first review the previous work which this work is built upon, then will detail the singularity problem and propose a grouping procedure using Dulmage-Mendelsohn decomposition. This grouping procedure allows us to detect the number and type of additional precise constraints needed for resolving splice variants. We then provide a general Bayesian framework for incorporating imprecise constraints, and construct a probe responsiveness model using the probe sequence to provide such constraints.

### Previous Model

Wang *et al*. [36] developed the following model to estimate variant concentrations by relating transcript concentrations to the observed probe intensities:

(1)

**Figure 9:**
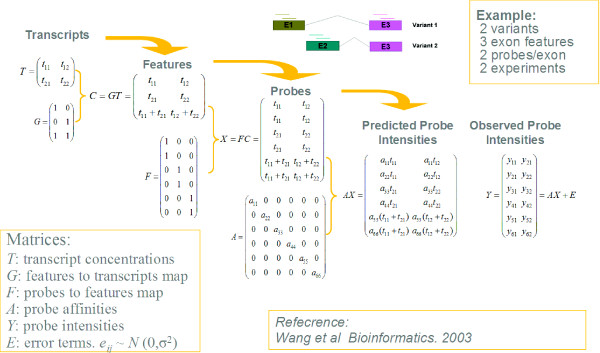
**Matrix representation of Wang et al.'s model **[36].

The observed probe intensities are given by a *p-by-x *matrix ***Y ***= [*y*_*ij*_], where *y*_*ij *_is the intensity of probe *i *for experiment *j*. ***A ***= [*a*_*ii*_] is a *p-by-p *diagonal matrix with *a*_*ii *_representing the probe responsiveness for probe *i*. ***F ***= [*f*_*il*_] is a *p-by-q *matrix with values 0 or 1, where *p *is the total number of probes, *q *is the total number of features; *f*_*il *_equals 1 if probe *i *belongs to feature *l*, and 0 otherwise. The relationship between features and transcripts is represented by a *q-by-t *matrix ***G ***= [*g*_*lk*_], referred to as the gene structure matrix, containing binary values of 1 or 0, where *g*_*lk *_= 1 means feature *l *is present in transcript *k*, while *g*_*lk *_= 0 means this feature is absent. The transcript concentrations of a given gene in all experiments are represented by a *t-by-x *matrix ***T ***= [*t*_*kj*_], where *t*_*kj *_represents the concentration of transcript *k *in experiment *j*. See Figure 9 for an illustration of these matrices. The observed probe intensities ***Y ***are noise contaminated signals, with experimental error matrix denoted by ***E ***= [*ε*_*ij*_]. Heteroscedasticity was introduced in the model by assuming *ε*_*ij*_~N (0, *σ*^2^max(*y*_*ij*_, *C*)) for some properly chosen constant C. An additional scale constraint on  is added in fitting the model using Maximum likelihood estimation (MLE) to estimate the unknowns ***A ***and ***T***.

### The Non-uniqueness Problem

Typically, ***A ***and ***T ***have non-unique solutions for many given gene structures ***G***. Consider, for instance, the following ***G ***with the second column a 'subset' of the first one in terms of the transcript composition:



This ***G ***matrix has full rank, yet the algorithm converges to multiple global minima with infinitely many solutions (see Figure 1 for an illustration). In addition, increasing the number of probes or changing the optimization criterion in the fitting procedure does not alleviate the problem.

Before going into technical details and concrete algorithm design, we explain why the above singularity constitutes an intriguing problem in practice and introduce our rough ideas for removing the deconvolution ambiguity in gene alternative splicing. First, note that this non-uniqueness is essentially different from the common issue of multiple local minima in nonconvex optimization. We will show that when the problem occurs, there exist infinitely many solutions of the target concentration matrix ***T ***all of which attain the global optimum but far from the true ***T***, as a result of some type of degeneracy in the gene structure matrix ***G***. This indicates that a macro-model relating transcript concentrations to observed probe intensities (like Li-Wong [34] or our previous model given by (1)) alone cannot resolve the non-uniqueness. It reflects the ill-posed nature of this deconvolution task: additional sources of information must be provided to remove the ambiguity and to identify the truth.

Naturally, we turn to probe sequences at the microscale and aim to build a probabilistic probe responsiveness model on sequence composition. However, to the best of our knowledge, none of such models is perfect, due to the fact that probe responsiveness is a sophisticated biochemical process which is dependent on many other factors in addition to sequence composition. Therefore, incorporating these imperfect predictions from a micro-model into a macro-model is a challenging problem, and we must take a *statistical *point of view.

In short, additional constraints on ***A ***or ***T ***are needed to overcome the singularity. The following questions then arise: How much information is sufficient? What type of constraints should we impose in the fitting procedure? In the next subsection, we provide a rigorous mathematical analysis to answer these questions.

### Solving the Singularity Problem Mathematically

Suppose ***G ***is known. We consider the problem of recovering ***A ***and ***T ***given

(2)

where ***A ***is a diagonal matrix.^1 ^Assume ***G ***satisfies the following properties:(i) ***G ***is a 0–1 matrix; (ii) ***G ***has full column rank; (iii) ***G ***has no zero rows. Define the following solution spaces:



Assume the decomposition problem (2) is solvable in *W*_1 _or *W*_2_. The following two theorems establish the relationship between the separability of ***G ***and the uniqueness (up to a global scaling constant, which is implicitly assumed throughout this section) of solution.

**Theorem 1**. *(Necessity) Suppose Problem (2) is solvable in W*_1 _*under the above setup. Then the solutions can not be unique, as long as there exists some nonsingular ****C ****such that ****M ***= ***GC***^-1 ^*is separable*.

*Here*, ***M ****is separable in the sense that there exist some row permutations ****P ****and column permutations ****Q ****such that ****PMQ ****is block diagonal, say*, . *Note that the blocks are *not *necessarily square*.

The proof is simple. Let (***A***, ***T***) ∈ *W*_1 _be a solution to (2), ***M***^0 ^be a separable matrix for some nonsingular ***C***, and permutation matrices ***P***, ***Q***. That is,



Define ***Y***^0 ^= ***PY***, ***A***^0 ^= ***PAP***^*T*^, ***T***^0 ^= ***Q***^*T*^***C***T. We get

(3)

According to the block diagonal form of , write ***A***^0 ^as , and ***T***^0 ^as .

Define



for any *a*, *b *> 0. Then . The conclusion thus follows by noticing that ***C***^-1^***Q *** is continuous for positive (*a*, *b*) and *W*_1 _is open.

The following sufficiency theorem states that we only need to study one special ***M ***for uniqueness. This has great advantage in practice.

**Theorem 2**. *(Sufficiency) Suppose Problem *(2) *is solvable in W*_2_. *Then if a 'maximally orthogonalized' ****M ****(as will be clear below) is nonseparable, and ****T ****is of full row rank, then the solution is unique in W*_2_.

*Here ****M ****is generated in the following way: apply as many as possible column subtractions to ****G***, *until we get a nonnegative ****M ***= ***GC***^-1 ^*with the maximum number of zeros*. ***M ****is referred to as a 'maximally orthogonalized' version of ****G***.

Before proving the theorem, we introduce a simple lemma.

**Lemma**. *If ****MX ***= ***MY ****and ****M ****has full column rank, then ****X ***= ***Y ***.

In fact, we can get nonsingular  = [***M M*_⊥_**] by expanding *M *since it has full column rank. Then



Let (***A***, ***T***), (***B***, ***S***) ∈ *W*_2 _be two solutions. Note that in this case the elements of ***M ***are either 0 or 1, and ***C ***is nonnegative. Introduce the same symbols as before: ***M***^0 ^= ***PMQ***, ***Y***^0 ^= ***PY***, ***A***^0 ^= ***PAP***^*T*^, ***T***^0 ^= ***Q***^*T*^***CT***, ***B***^0 ^= ***PBP***^*T*^, ***S***^0 ^= ***Q***^*T*^***CS***, and assume ***M***^0 ^is in the following echelon form without loss of generality:

(4)

Denote by *l*_*j *_the row index of the first 1 in the *j*-th column of ***M***^0^, 1 ≤ *j *≤ *q*, where *q *is *rank*(***M***^0^) (or the number of columns of ***M***^0 ^by assumption). Let ***D ***= ***A***^0^. We need to show ***D ***∝ ***I***; if so, the theorem follows by the lemma.

Suppose for the moment *diag*(***D***) consists of two distinct values *a*, *b*, and ordered in the way of  with a ≠ *b*, and *n*, *m *≥ 1.

If *n *≥ *l*_*q*_, write ***M***^0 ^as  according to the block form of ***D***. It is easy to see from (4) that  is full column rank. So



The last equivalence is by the lemma.

On the the other hand,  must have at least one positive entry, otherwise



would have zero row(s). Put one such index to be (*i*, *j*). Then the *j*-th row of ***T***^0 ^is **0**, and so is a certain row of ***QT***^0^. Moreover, to make sure ***T ***= ***C***^-1^***QT***^0^is nonnegative, the row subtractions ***C***^-1 ^must not change this particular row. Accordingly, ***T ***also has zero row(s), which contradicts the definition of *W*_2_.

If *n *<*l*_*q*_, say *l*_*j*-1 _≤ *n *<*l*_*j *_for some *j *>1. Write ***M***^0 ^as , with the number of rows of ***X ***or  being *m *- (*l*_*j *_- *n *- 1) ≙ *m'*, and , , , accordingly. For convenience, we use ***Z***(*i*_1_, ⋯, *i*_*k*_; *j*_1_, ⋯, *j*_*k*'_) to denote the submatrix of ***Z ***made by the entries located at the *i*_1_-th, ⋯, *i*_*k*_-th rows and the *j*_1_-th, ⋯, *j*_*k*' _-th columns, and *Z*(*i*_1_, ⋯, *i*_*k*_;:) the submatrix by taking out the *i*_1_-th, ⋯, *i*_*k*'_-th rows of ***Z***. From



we have



Recall that [***X ***] is a submatrix from the echelon form of ***M***^0 ^(see (4)), composed of *q *- *j *+ 1 blocks: ***M***^0^(*l*_*j*_, ⋯, *l*_*j*+1 _- 1;:), ***M***^0^(*l*_*j*+1_, ⋯, *l*_*j*+2 _- 1;:), ⋯, and ***M***^0^(*l*_*q*_, ⋯, *n *+ *m*;:).

Let us consider the *l*_*j *_block ***M***^0^(*l*_*j*_, ⋯, *l*_*j*+1 _- 1;:) first. We can write it as [***Z***_*j *_*e*_*j *_0], where *e*_*j *_is the *j*-th column of this block. Let ,  be the *j*-th row vectors of ***T***^0^, ***S***^0^, respectively. Then from



We get the basic equations:

(5)

(6)

Discuss the following three cases:

1. There exist two rows (*k*_1_, *k*_2_) in [***Z***_*j *_*e*_*j*_] such that ***Z***_*j*_(*k*_1_;:), ***Z***_*j*_(*k*_2_;:) are different, but *e*_*j*_(*k*_1_) = *e*_*j*_(*k*_2_). Then it is not difficult to learn from (6) that the rows of  are linearly dependent (details omitted). And so ***T ***= ***C***^-1^***QT***^0 ^can not have full row rank.

2. There exists some row *k *in [***Z***_*j *_*e*_*j*_] such that *Z*_*j*_(*k*,:) has at least one 1, but *e*_*j*_(*k*) = 0. Observing the entries of (*b *- *a*) all have the same sign in (6), we immediately obtain (*k*';) = **0 **for some *k*'. By a similar argument as in the case *n *≥ *l*_*q*_, ***T ***must have a zero row, which makes a contradiction again.

3. Therefore, [***Z***_*j *_*e*_*j*_] can only be made up of identical rows apart from the zero rows. From the construction of the 'maximally orthogonalized' ***M***^0^, given any column index *β*: 1 ≤ *β *≤ *j *- 1, there must exist some row index a such that ***Z***_*j*_(*α*;*β*) = 0, *e*_*j*_(*α*) = 1. Therefore, the rows of the [***Z***_*j *_*e*_*j*_] are either [0 ⋯ 0] or [0 ⋯ 0 1]. But then ***Z***_*j *_= **0**, from which it also follows that .

For the next block ***M***^0^(*l*_*j*+1_, ⋯, *l*_*j*+2 _- 1;:) that can be written as



due to , it is easy to verify that the basic equations become



And ***Z***_*j*+1 _= **0**,  by similar reasoning. Continue this process till block the *l*_*q*_th block; we will finally obtain ***X ***= **0**, which means ***M***^0 ^is separable. In summary, *a *= *b *must be true.

In fact, the above discussions can generalize to *diag*(***D***) containing multiple distinct values which are not necessarily ordered. We omit the proof details as the derivations are similar. (In fact, the key is still the 3-case discussions; yet note the the contradiction in case 2 would be made regarding the full row-rankness of ***T***. In the end we get a block diagonal matrix which is again separable.) Theorem 2 is now proved.

### Intuition of the Theorems

In the following, we will give an intuitive explanation of the theorems using a few simple examples.

First, it is easy to see that if there exists some nonsingular ***C ***such that ***M ***≙ ***GC ***is 'separable', that is, ***M ***can be written in a block diagonal form like



where all blocks are not necessarily square, then the solution, if exists, must be non-unique. So our task is to prevent such ***M ***from being separable. Moreover, our sufficiency theorem states that we only need to consider one particular ***M ***that is 'max-orthogonal' (described in Theorem 2): if it is nonseparable, *and ****T ***has full column rank, then the solution is essentially unique. The full rank requirement can not be dropped in general, but is possible for some special cases.

Constraints can be imposed on ***A ***or ***T ***or both. In this work, unless otherwise stated, we construct constraints on ***A***. Basically, the number of rows of blocks of the finest block diagonal form of the 'max-orthogonalized' ***M ***is the minimum number of constraints we need, and the effective constraints should be between-block quantities to overcome the factorization singularity.

Now we apply our theorems to some special examples. First, consider



from which we have



Although this structure is a valid biological example, it is impossible to uniquely discern the transcript concentrations. Intuitively, this is due to the ambiguity caused by the the third transcript and the conjunction of the first two transcripts. But our grouping analysis is able to tell what type of information and how much we need to overcome this problem for any complex ***G***.

Next let us take a look at the alternatively spliced gene structure in Shai *et al*. [37]. The cassette exon structure can be expressed as



Based on our theorems, it is easy to see that the solution is unique.

Finally, we point out the fact that the rank constraint can not be dropped in general; as an example, consider



where



***G ***is nonseparable but *rank*(***T***) = 1. The solutions are not unique. This conclusion provides some guidance in experiment design. As a conservative consideration in selecting the transcripts to do microarray experiments, we want ***T ***to have full row rank, to reduce the problem of deconvolution singularity to the issue of ***G***-separability only. On the other hand, given a particular ***G***, through studying which of the three cases the 'max-orthognalized' ***M ***falls into, we may have a broader choice. For example, if each individual probeset corresponds to a different transcript, i.e., the ***G ***in Theorem 2 is given by ***F***·***I ***or ***F***, then case 1 does not exist, and the rank constraint can be dropped; the same conclusion is true for ***G ***with only 2 columns (2 variants).

### Grouping Procedure in Practice

In practice, to obtain the finest (irreducible) block diagonal structure of ***M***, we use the *Dulmage-Mendelsohn decomposition *[40] which arose in finding strong Hall components in graph theory. The whole procedure is summarized as follows.

#### Grouping Procedure

1. Basis transformation.

(a) Apply column subtractions to ***G ***until we get a 'max-orthogonal' ***M ***with ***GC ***= ***M ***(see Theorem 2 for the detailed explanation).

(b) Remove the zero columns of ***M***.

2. Find the sparse structure (or the finest block diagonal form) of ***M***.

(a) Compute ***N ***= ***MM***^*T*^.

(b) Find the sparse structure of ***N ***using Dulmage-Mendelsohn decomposition. Denote by ***P ***the corresponding row permutations.

(c) Applying ***P ***to ***M***, obtain the column permutations ***Q ***such that ***PMQ ***is block diagonal. Each diagonal block (not necessarily square) defines an individual group as an outcome. Record the group indices from ***P***, ***Q***.

After the procedure, the probes are classified into a few groups. As mentioned earlier, the number of groups is the minimum number of constraints required. Effective probe-responsiveness constraints should be imposed *between *groups.

It is worth mentioning that too many constraints do not necessarily lead to a better solution. This is especially true when the constraints are on ***A ***but ***A ***is poorly estimated. For example, incorporating every responsiveness estimate as an independent constraint into the deconvolution process, though providing enough information to remove the non-uniqueness, is very sensitive to the estimation errors in ***A***. In contrast, group-based constraints offer just enough information to deal with the singularity, and the group means of the estimated responsiveness have smaller error and provide a more stable recovery of ***T ***in the end.

### A Bayesian Framework for Estimating Splice Variant Concentrations

**Figure 10:**
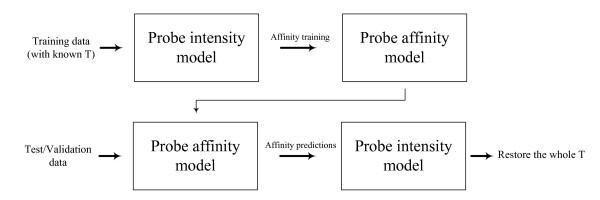
**Procedure outline for estimating transcript concentrations**. The probe sequence model is trained supervisedly on some data with ***T ***available. Then predict the (grouped) probe responsiveness on the new dataset to help recover all concentrations via the probe intensity model.

Our idea for resolving deconvolution ambiguity in gene alternative splicing is as follows. First use experimental data with known ***T ***to estimate ***A***. Train a probe responsiveness model on sequence composition in a *supervised *fashion. Next for all probes in the target microarray data, predict their responsiveness based on their sequences. Finally construct group constraints and use this information to fit a probe intensity model to get a unique solution of all transcript concentrations. The procedure is shown in Figure 10.

Unfortunately, according to our experiments, the predictions from any existing probe sequence-based model are found to be poor and so we adopt a Bayesian framework to include the standard errors. This framework combines a probe intensity model and a probe sequence model into one optimization function. We will address these aspects in detail below.

#### Probe Intensity Model

A regression model with additive errors, by Li and Wong [34] and Wang *et al*. [36], is used in our computation with modifications:

(7)

or in the scalar form of

(8)

Typically, *y*_*ij *_is PM_*ij *_- MM_*ij *_as in most literature. We propose a novel way of constructing the responses *y*_*ij*_

(9)

where  denotes the minimum intensity of the *i-th *probe in all experiments, and  the minimum intensity of the total array. Note that it does not require MM probes. In principle, this PM correction assumes the array has at least one experiment with no signal (*T*_*ij *_= 0 for some *i*, *j*) which is true for nearly all practical microarray data. Our experiences show that the new responses given by (9) have an advantage over the usual way of subtracting MM from PM signals, which may have something to do with the founding that the hybridization sources can be different for many PM and MM probe pairs [41].

We construct a *weighted *additive model, denoted as WAM, to avoid taking the log transformation of the *y*_*ij *_(possibly negative). Let ∈_*ij*_~*N *(0, ). If there are MM probes available, let  ∝ PM_*ij *_· MM_*ij*_, which can be seen as a result of the positive correlation between PM probes and paired MM probes. In fact, it can be justified from the Affymetrix PLIER model [42] by applying the Delta method to it. If there are no MM intensities, we simply put . Either way is simple and successful; they show a significant improvement over the original additive models by Li and Wong [34] and Wang [36].

#### Probe Responsiveness Model

**Figure 11:**
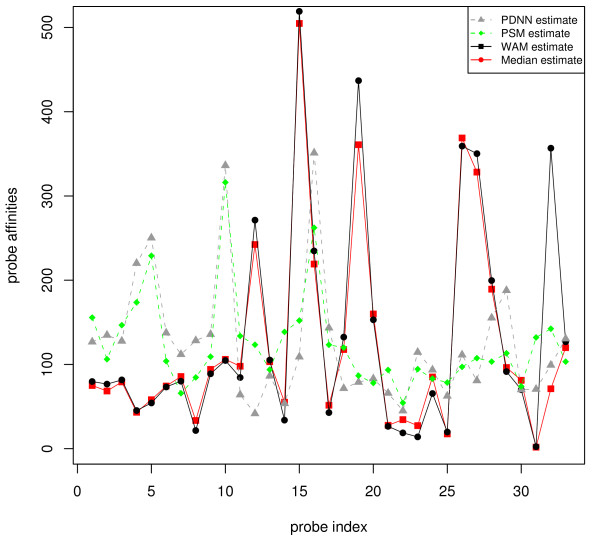
**Model training: supervised vs. unsupervised**. The figure gives a comparison of some response estimates. The first two are both *unsupervisedly *trained, with ***A ***and ***T ***unknown, from the PDNN model [43], and the probe selection model (PSM) [44], respectively. The WAM estimate is obtained by estimating ***A ***(with ***T ***known) from our model (7); a robust median estimate (the median of the ratios of ***Y ***to ***FGT***) is also plotted. Note that the first two deviate from the last two which are trained in a supervised manner.

First, consider the nearest neighbor model – PDNN by Zhang *et al*. [43], and a physical probe selection model (PSM) by Mei *et al*. [44]. Both models train the sequence-related parameters in an 'unsupervised' way, because no information from transcript abundances (***T***) is exploited in the training. We found these probe responsiveness estimates can be far away from the truth. Using spike-in data, we can compare them to the more trustworthy estimates derived *supervisedly *by using the ***T ***in the model fitting, as shown in Figure 11.

As we can see from the figure, the response estimates from PDNN and PSM, though similar to each other, seem to deviate a lot from the 'truth' (which can be learned well on the spike-in data); indeed, the unsupervised nature of the trainings makes it a very difficult task to estimate both ***A ***and ***T ***at the same time. In contrast, by taking the information from ***T ***into account, supervised methods, such as the robust median estimator or our WAM below, only need to estimate ***A ***in the fitting procedure, and is more accurate and efficient. Correspondingly, for spike-in data with ***T ***available, the supervised methods are far better than unsupervised methods in training a probe sequence model.

Motivated by the physical models proposed by Naef and Magnasco [45] and by Wu *et al*. [46], we build the following probe responsiveness model on a 25-nucleotide-long probe sequence .

(10)

where ∈ ~*N*(0, *σ*^2^), *b*_*i *_is one of the four nucleotide bases (A, T, C, G) at position *i *of a probe. *SP*^(*A*)^, *SP*^(*G*)^, *SP*^(*C*) ^and *SP*^(*T*) ^are four unknown natural cubic spline functions. They reflect the nucleotide difference and the position-dependent effect in modeling the probe responsiveness. It is worth pointing out that there is an essential difference between our model and GCRMA [46]. GCRMA models probe responsiveness in the sense of *non-specific *binding (NSB), corresponding to ***T ***= 0, while ours characterizes the probe responsiveness for *specific *targets in a particular dataset, which is meaningful to the problem we are trying to solve.

**Figure 12:**
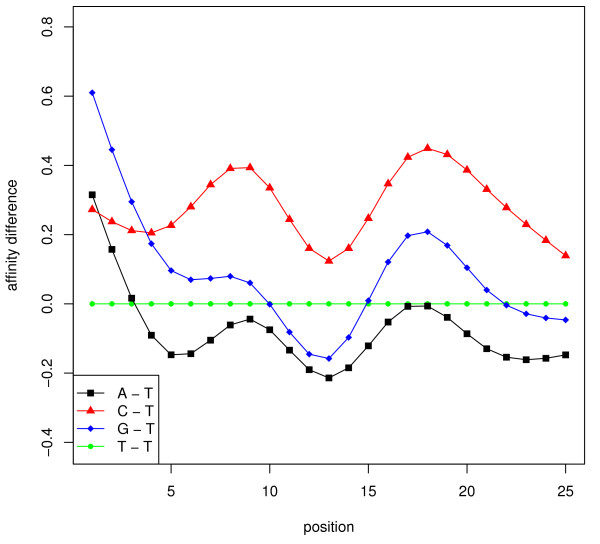
**Probe responsiveness curves**. Four position functions are shown to reflect the nucleotides' difference in responsiveness. The baseline is 'T' on 25 positions of every probe. The three curves correspond to the probe responsiveness change if replacing 'T' by 'A', 'C', and 'G' respectively. These functions are estimated using smoothing splines.

Clearly, there exists redundancy in the sequence model (10) because A, T, C, G all appear in it and each sequence is of fixed length. To avoid over-parametrization and remove ambiguity, we take *SP*^(*T*) ^as a constant function, that is,  is chosen as the baseline, and now the value of  represents the response change if we replace the nucleotide at the *i-th *position from T to *b*_*i *_(= A, C, G). To specify the natural cubic splines, we select 7 knots at positions 1, 5, 9, 13, 17, 21, 25, which give the best adjusted *R*^2 ^value empirically. So there are 7 × 3 + 1 = 22 unknowns. Figure 12 demonstrates the spline functions trained on the HG-LS data, from which the nucleotide difference and position dependency in modeling the probe responsiveness are clearly seen. These (specific-binding) probe responsiveness are different than non-specific binding curves [45,46].

#### Bayesian Priors and Optimization

**Figure 13:**
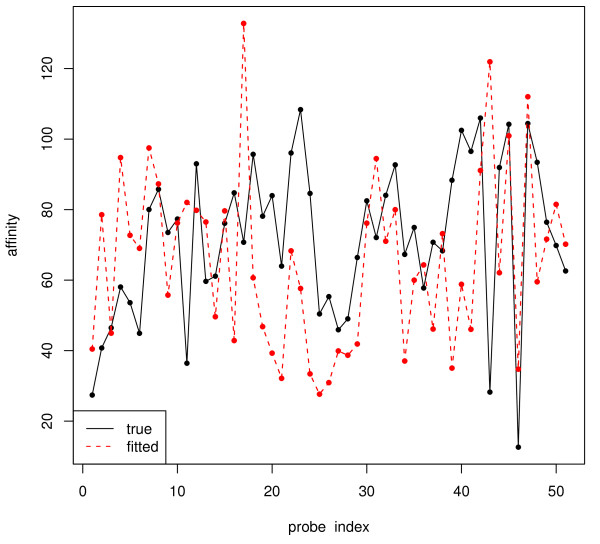
**Trained probe responsiveness on HG-LS data**. We present 50 probes chosen at random. The true probe responsiveness is in solid lines while the value fitted with supervised training is in dashed lines. The fitted values deviate a lot from the true for most probes, which indicates poor goodness-of-fit of the probe responsiveness model. It is worth mentioning that most papers choose to display similar quantities on log scale. But to have a small error in ***T***, ***A ***should be well predicted even on the original scale.

Theoretically speaking, after fitting the responsiveness model, we are able to predict the response for any probe with known sequence composition to a given concentration; group constraints can be easily constructed to resolve the singularity problem in the recovery of all transcript concentrations. The practical difficulty is, however, the trained responsiveness model does not fit well enough in general. Figure 13 below shows how poor the estimates can be, which is unfortunately true for any existing probe response model to-date. It is worth mentioning that to ensure the error in estimating ***T ***is small, the prediction for ***A ***should be accurate on the original scale instead of the log scale as most other methods chose to display their fitted variables.

To solve this problem, we use a Bayesian approach by including the uncertainties of probe responsiveness predictions into the model. For each probe group *G*_*k*_, we make a prediction, denoted by *Pred*_*k*_, for the geometric mean of probe responsiveness, with the standard error given by *se*_*k*_. Then incorporate 'fuzzy' group constraints into the fitting procedure of the probe intensity model, by minimizing the following function *L*:

(11)

**Figure 14:**
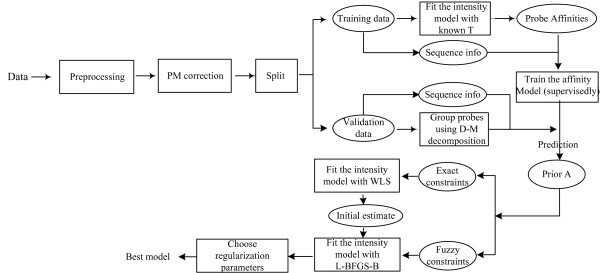
**A Bayesian framework for building transcript concentration estimation model**. The sequence based model is trained on the training data in a supervised way. Then for the validation data, group-constraints are constructed from the predicted probe responsiveness to remove deconvolution ambiguity as discussed earlier. 'Fuzzy' constraints can be considered in fitting a probe intensity model with standard errors included (see (11)). During the optimization, the exact group-constraints with no standard errors serve as an initial estimate.

Minimizing (11) amounts to solving the maximum likelihood estimation of a Bayesian model with Gaussian priors. The regularization parameter *λ *is determined by validation. Figure 14 summarizes the complete Bayesian procedure of building the model.

### Related Work

Recently, a work related to reconstruction non-uniqueness has appeared [47], where Lacroix *et al*. studied a transcriptome reconstruction problem in sequencing experiments. Under the *exact information *hypothesis (which may not be realistic at the present time as noted by the authors), they considered linear systems and introduced the notion of interchangeable sets to identify the possible singularity theoretically. Mathematically speaking, this is a much simpler problem compared to resolving deconvolution ambiguity in microarray data analysis. In fact, Lacroix *et al*. studied the model of ***y ***= ***Gt ***with ***y***, ***t ***being vectors, while we investigate a higher order equation with errors–see equation (7) for example. In [47], nonuniqueness only arises from the rank deficiency of ***G***, but even a ***G ***of full rank, like the subset gene structure or an identity ***G***, leads to infinitely many solutions in our setup due to the existence of the unknown ***A ***which represents the probe responsiveness. To address such problems, we perform a rigorous mathematical analysis and take a statistical point of view in the algorithm design. We also go a step further by proposing to use probe sequence information to resolve the reconstruction ambiguity, in addition to identifying all possible singularities of the model using our grouping procedure introduced earlier.

Another related work is [39] in which Anton *et al*. used nonnegative matrix factorization (NMF) to fit Wang *et al*.'s model (1) in optimization. NMF is a class of algorithms in multivariate analysis. As a matter of fact, our fitting procedure in implementation is an example of NMF, too. The difference is that our approach has more statistical flavor. In microarray data analysis, we are able to build a statistical model by relating transcript concentrations to the observed probe intensities, like Li and Wang [34] or our model, with heteroscedastic errors. With such an explicit error distribution available, the log-likelihood naturally acts as a specific optimization criterion which tends to yield better estimation accuracy than a general purpose divergence function like K-L entropy used in their paper. Moreover, it is worth pointing out that [39] does not fully tackle the deconvolution ambiguity problem as addressed in this paper. We have mentioned when introducing the non-uniqueness problem that even when the gene structure is known, there may exist infinitely many *global *minima far from the true ***T***, which is essentially different than the common issue of multiple local minima in optimization as NMF. The nature of this nonuniqueness determines additional source of information must be provided to remove the ambiguity and to identify the truth. Nevertheless, this serious problem is more or less masked out by the well-known fact that NMF possesses too many local optima.

In the experiments performed in the **Results **Section, we include the SPACE algorithm by Anton *et al*. [39] for comparison purpose.

## Authors' contributions

YS performed the mathematical analysis and the statistical modeling and was responsible for the algorithm design and the programming. HW conceived of the study, participated in its design and provided guidance. YS and HW drafted the manuscript. EH helped building the model and writing the manuscript. All authors read and approved the final manuscript.

## Note

^1^It is easy to see dropping F from the original formula has no effect on the following analysis.
